# Understanding Risk Factors for Suicide Among Older People in Rural China: A Systematic Review

**DOI:** 10.1093/geroni/igae015

**Published:** 2024-02-17

**Authors:** Quan Zhang, Shenao Li, Yijin Wu

**Affiliations:** School of International Affairs and Public Administration, Ocean University of China, Qingdao, Shandong, People’s Republic of China; School of International Affairs and Public Administration, Ocean University of China, Qingdao, Shandong, People’s Republic of China; Center for Geriatric Healthcare Services and Health Education, Qufu Normal University, Rizhao, Shandong, People’s Republic of China

**Keywords:** Suicidal behavior, Suicide detection, Suicide screening, Suicide prevention

## Abstract

**Background and Objectives:**

In China, rural older adults face a significantly heightened risk of suicide. However, there has been no comprehensive review of the literature examining the risk factors associated with suicide among older people in rural China. Therefore, a comprehensive understanding of risk factors for this phenomenon among rural older people must be gained. We conducted a systematic literature review on risk factors for suicide among older people in rural China.

**Research Design and Methods:**

Seven English electronic databases (PubMed, EMBASE, PsycINFO, Cochrane, CINAHL, ScienceDirect, and Web of Science) and 3 Chinese electronic databases (CNKI, CQVIP, and Wanfang) were searched for peer-reviewed articles published in English or Chinese, from inception to July 25, 2022. For data collection, scientific strategies were used for searching and selecting literature within the electronic databases. The collected data were then synthesized using the thematic analysis method. The study was conducted under PRISMA 2020 guidelines.

**Results:**

The final analysis included 16 studies. The identified risk factors were categorized under 6 themes: navigating the challenges of illness, unmet basic needs, experiencing abuse from children, feelings of loneliness, negative life events, and altruistic motivation to benefit children.

**Discussion and Implications:**

Multiple factors affect suicide among older people in rural China. This invaluable information can be used to develop targeted prevention strategies particularly relevant to this age group.

**Translational Significance:** The findings of this research have profound implications for implementing coping mechanisms to effectively mitigate the high rates of suicide in rural areas. To address this pressing issue, it is crucial for healthcare professionals to prioritize early identification and intervention, by integrating suicide risk assessments into routine medical evaluations for older adults residing in rural regions. Adopting a synergistic approach that involves the active participation of the Chinese government, nonprofit organizations, and familial networks is essential. This collaborative effort will enable the delivery of tailored services that strengthen social support networks for at-risk older adults in rural areas.

Suicide is a complex social issue transcending geographic boundaries (Bachmann, 2018; Turecki & Brent, 2016). Despite the global suicide rates declining gradually since 1990, the overall number of suicide deaths remains alarmingly high (Naghavi, 2019; Yip et al., 2022). The World Health Organization (2021) reported a 36% reduction in age-standardized global suicide rates between 2000 and 2019. Yet, an astonishing 703,000 suicides occur annually. Furthermore, a notable disparity in suicide rates has been observed between urban and rural areas in many countries (Casant & Helbich, 2022). For instance, urban–rural differences in Germany’s suicide rates were confirmed by regression models, rural areas have higher suicide rates (range from 12.6 and 13.2 deaths per 100,000 inhabitants) compared with urban areas (range from 11.0 to 11.6 deaths per 100,000 inhabitants; Helbich et al., 2017). Similarly, a 2018 comprehensive survey conducted in the United States revealed that rural suicide rates (19.4 deaths per 100,000 inhabitants) were higher than urban suicide rates (13.4 deaths per 100,000 inhabitants; Pettrone & Curtin, 2020). This incongruity in suicide rates between urban and rural areas has long been established in China and continues to persist today (Jiang et al., 2018; Phillips et al., 2002). In a study conducted in China’s Shandong Province, the rural suicide rate (19.0 deaths per 100,000 inhabitants) was 3.5 times greater than the urban suicide rate (5.3 deaths per 100,000 inhabitants) between 2006 and 2010 (Sun et al., 2013). In another 2015 survey, the rural suicide rate was twice as high as the urban suicide rate in China (Jiang et al., 2018). These findings align with the findings reported in the China Health Statistics Yearbook. This yearbook reported that the urban suicide rate in China was 4.31 deaths per 100,000 inhabitants, whereas the rural suicide rate was 7.09 deaths per 100,000 inhabitants in 2021 (National Health Commission of China, 2022).

In rural China, the suicide rate among older persons is higher than that among young people (Liu et al., 2015; Yip et al., 2008). The statistics from 2021 reveal alarming differences in suicide rates among various age groups in rural China. For young and early middle-aged adults, aged 20–25 years, 25–30 years, 30–35 years, 35–40 years, and 40–45 years, the rates range from 3.66 to 4.14 deaths per 100,000 residents (National Health Commission of China, 2022). However, among older adults, aged 65–70 years, 70–75 years, 75–80 years, 80–85 years, and those over 85 years, the rates are even more concerning, ranging from 14.39 to 38.58 deaths per 100,000 residents. The risk of suicide is the highest among older people in rural China (Dummer & Cook, 2007; Sun & Jia, 2014). Consequently, suicide among rural older individuals has emerged as a substantial social issue in China (Chen, Mo, et al., 2022; Zhou et al., 2021).

The issue of suicide among older people in rural China has been extensively investigated. In investigating suicide-associated temporal patterns, Wu et al. found that winter is the predominant season for suicide among older individuals in rural China, with most incidents occurring at night (Wu et al., 2009). Zhu et al. explored the suicide methods employed by older people in rural China. Their findings revealed that ingestion of pesticides (51.7%) was the most common method, followed by hanging (39.3%), ingestion of nonpesticide poisons (3.3%), jumping from heights (1.2%), and wrist cutting (0.8%; Zhu et al., 2019).

Other studies have focused on identifying risk factors for suicide among older people in rural China. A substantial number of studies have investigated the factors contributing to this occurrence among older adults in rural China. These studies have offered diverse perspectives and valuable insights into comprehending this issue. However, to the best of our knowledge, literature on risk factors for suicide among older people in rural China has not been comprehensively reviewed. Therefore, this study aimed to fill this gap by conducting a systematic review of published articles obtained through electronic databases. By synthesizing the data gathered, we approach risk factors for suicide among older adults in rural China with different thematic focuses.

## Method

### Study Design

We conducted a systematic review investigating the risk factors for suicide among older adults in rural China. A systematic review is a robust and reliable method for conducting literature reviews. It is a replicable, transparent, and unbiased approach for interpreting existing studies systematically and objectively by using the classification process of coding and identifying themes (Aromataris & Pearson, 2014; Boell & Cecez-Kecmanovic, 2015). Using a systematic review, we could summarize diverse risk factors for suicide among older individuals in rural China. Because of its capacity to systematically interpret the literature and elucidate the richness and uniqueness of the data, a systematic review has been extensively used for synthesizing the risk factors for suicide among older people globally (Beghi et al., 2021; Conejero et al., 2018; Fernandez-Rodrigues et al., 2022; Holm & Severinsson, 2015).

We employed a methodological approach, pioneered by Aromataris and Pearson (2014), that consists of five stages. In the first stage, the research question is identified as it could guide our data collection efforts. Our research question was “What are the multiple risk factors for suicide among older people in rural China?” The second stage involves a comprehensive search for relevant studies. A scientific strategy was employed to ensure that the literature retrieved addressed the research question. In the third stage, studies were selected and their qualities were assessed. A systematic approach was used to select literature relevant to our research question. We used the Mixed Methods Appraisal Tool (MMAT) to appraise the quality of the included studies. The fourth stage involves data extraction and synthesis. Themes and subthemes were developed using a narrative synthesis method. In the fifth stage, the findings were interpreted and recommendations were provided to guide practice.

### Data Collection

#### Literature search

A thorough literature search was conducted to explore the topic of suicide, including completed suicide, and attempted suicide, among older people in rural China. Completed suicide refers to an act in which an individual successfully ends their own life through self-harming behavior, whereas attempted suicide refers to an act in which an individual attempts to end their own life through self-harming behavior but does not succeed (De Leo et al., 2001; Parra Uribe et al., 2013). Seven English electronic databases (PubMed, EMBASE, PsycINFO, Cochrane, CINAHL, ScienceDirect, and Web of Science) and three Chinese electronic databases (CNKI, CQVIP, and Wanfang) were utilized. The search spanned from the inception of databases to July 25, 2022. The search strategy was formulated using the PICo template (Table 1; Pettigrew & Roberts, 2006). The literature search terms (MeSH terms) used to retrieve relevant studies for each database is as Supplementary Material 1. All studies retrieved were exported into reference management software NoteExpress for easy management.

**Table 1.** The Search Strategy Using the PICo Template

Search strategy	PICo term	Literature search terms (MeSH terms)
Population	Elderly people	“elderly” OR “elder” OR “aged” OR “older” OR “senior” OR “old people” OR “old man”
Phenomenon of interest	Suicide behavior, including completed suicide, and attempted suicide	“suicide” OR “suicides” OR “suicidal” OR “self-murder” OR “self-slaughter”
Context	Rural China	“rural” OR “countryside” OR “village” OR “peasant” OR “farmer” OR “farmers” OR “villager” OR “villagers” AND “China” OR “Chinese”

#### Inclusion/exclusion criteria

Inclusion and exclusion criteria were developed to define the study scope and determine the studies to be included or excluded. These criteria were based on factors such as the target population, phenomenon of interest, study contest, publication type, research method, methodological rigor, sufficiency of findings, language of publication, and timespan. Table 2 details the inclusion and exclusion criteria.

**Table 2.** Inclusion and Exclusion Criteria

Criteria	Inclusion criteria	Exclusion criteria
Population	Older people (age ≥ 60 years)	Nonolder people (age < 60 years)
Phenomenon of interest	Suicide behavior, including completed suicide, and attempted suicide	Nonsuicide behavior, including suicide ideation, suicidal thoughts, suicidal intent, suicide prevention, suicide reduction, self-harm, nonsuicidal self-injury, suicide-related hospitalization, and suicide and social change
Context	In rural China	Not in China, not in a rural area
Type of publication	Research papers and peer-reviewed journals	Nonresearch articles, including letters, editorials, comments, correspondence, abstracts, guidance, news, errata, etc. Not published in a peer-reviewed journal
Research method	Empirical studies including qualitative, quantitative, and mixed-method studies	Nonempirical studies, including systematic review, meta-analysis, critical studies, editor’s introduction, etc.
Rigor of methodology	Clear report of the analytic procedure	No clear report of the analytic procedure
Sufficiency of findings	Sufficient results (results that provide rich information to the review question)	Not sufficient results
Languages	Studies published in English or Chinese language	Studies not in English or Chinese language
Time span	From inception until July 25, 2022	After July 25, 2022

#### Literature selection

**Figure 1.**
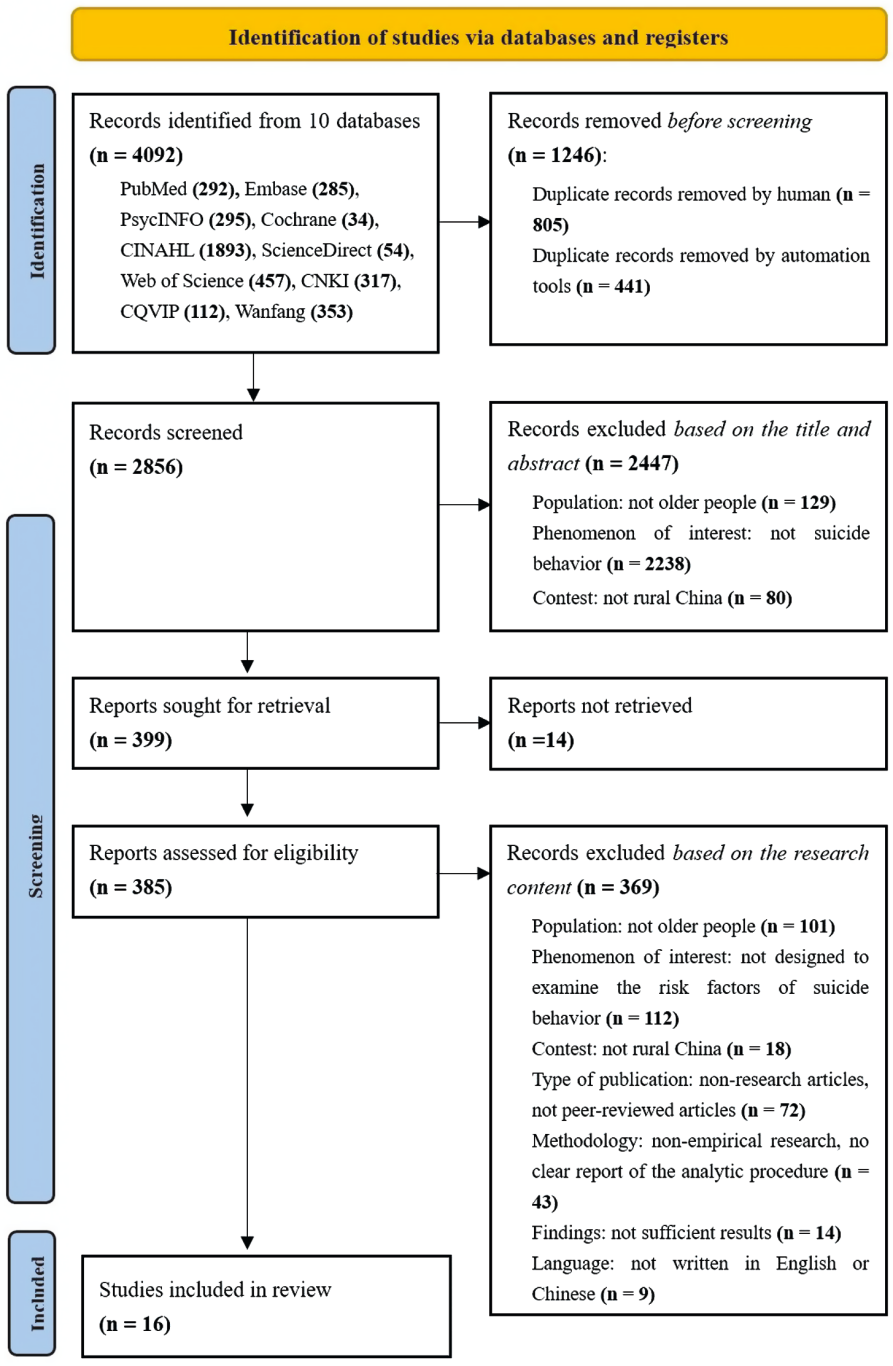
Flow chart of the literature identification and selection process.

The search process generated a collection of publications, which were selected following the three-step process proposed by Lockwood et al. (2015) (Figure 1). Initially, 4,092 articles were obtained. The title of each article was carefully reviewed to identify and remove duplicate entries. Accordingly, 1,246 duplicates were excluded during this stage. Subsequently, 2,846 articles were further screened based on their titles and abstracts. The titles and abstracts of 2,247 articles revealed that these articles were clearly outside the study scope and so were excluded. Of them, 129 records did not focus on older people. Furthermore, 2,238 records exhibited no strong correlation with the research question in this study, such as suicide thoughts and ideation (Poteat et al., 2020), suicide prevention (Sulyok & Walker, 2020), suicide reduction (Hoque et al., 2021), nonsuicidal self-injury (Bozkurt et al., 2021), suicide risk (Scott et al., 2021), and suicide-related hospitalization (Xie et al., 2021). Additionally, 80 records had a study context unaligned with rural China. As part of the third step, the full texts of the remaining 399 articles were carefully reviewed to determine if they complied with the inclusion and exclusion criteria. Consequently, 16 studies met the inclusion criteria for the present study. All selection process stages were independently conducted by two researchers (QZ, SL). In case of any disagreements at any stage, a third reviewer (YW) was consulted to achieve consensus (Khan et al., 2003).

### Quality Appraisal

Using MMAT, QZ and SL independently appraised the quality of the included studies (Pluye et al., 2011). This tool was intentionally designed as a checklist for concomitantly appraising the methodological quality of empirical studies included in systematic mixed-study reviews, including qualitative, quantitative, and mixed methods studies (Pluye et al., 2011). The MMAT (Version 2018) offers a set of criteria for screening questions for each study type, and accordingly, a score is assigned to each study. In case of any discrepancy during appraisal, a third researcher (YW) was consulted for resolution (Supplementary Material 2). Of note, no study was excluded based on its quality score (Harris et al., 2014).

### Data Extraction

Studies were extracted based on the following characteristics: authors, year, study quality (MMAT score), study design, study setting, data collection, participants, and key findings (Table 3). The first reviewer (SL) extracted data, which were re-checked by a second reviewer (QZ).

**Table 3.** Characteristics of the Included Studies

Study ^(MMAT)^	Study design	Setting	Data collection	Participants	Key findings
Bai et al. (2022)****	Mixed methods	Twelve counties of Shandong, Hunan, and Guangxi provinces, China	Key informant interview, structured interview, and semistructured interview	Key informants (next of kin, friends, neighbors, remote relatives) of 242 suicides in older adults	Physical illness, psychological distress, and interpersonal conflicts were the most prevalent risk factors for suicide among older adults in rural China.
Chen (2008)***	Qualitative	*Liwei* village in Anhui province, China	Key informant interview, participant observation, and filed note	Key informants (neighbors, relatives) of 19 suicide cases	The factors contributing to suicide among older individuals in rural China were identified as economic hardship, physical illnesses, and intra-generational conflicts.
Chen (2009) ****	Mixed methods	Six villages in Jingshan County, Hubei Province, China	Key informant interview and questionnaire	Key informants (friends, neighbors) of 206 suicide cases	The suicide rates among older people in rural China can be attributed to four primary factors: the desire to reduce the burden on children and extend children’s lifespan, distress caused by experiencing abuse from children, the despair resulting from the inability to obtain basic food, and the overwhelming sense of loneliness.
Chiu et al. (2012)***	Quantitative nonrandomized	Eleven villages in Mianyang City, Sichuan Province, China	Questionnaire	Rural older residents (*n* = 263)	A variety of factors have been associated with suicidal behaviors among rural older adults in China. These factors include being women, unmarried, having serious medical conditions, suffering from insomnia, facing financial hardships, having lower education levels, exhibiting depressive symptoms, encountering recent stressful life events, and reporting higher levels of life dissatisfaction.
Li et al. (2011)***	Quantitative nonrandomized	Rural communities in Hengyang County, Hunan Province, China	Questionnaire and semi-structured interview	Rural older residents (*n* = 1,040)	The incidence of suicide among older people in the rural communities in Hengyang county is influenced by several factors. Poor health conditions, exposure to highly stimulating negative life events, and depression have been identified as independent risk factors for suicide among rural older people.
Li (2017) *****	Qualitative	Four regions in Shandong Province, 1 region each in Hebei and Jilin Province, China	Key informant interview, questionnaire, and participant observation	Key informants (children, parents, spouses) of 156 cases of suicide in older adults, suicide attempters in older adults	Insufficient access to food, maltreatment by children, and relationship conflicts with children are the major risk factors for suicide among rural older adults. Moreover, negative life events such as the loss of children or neighborhood disputes may serve as catalysts for suicide among older adults. Notably, some older adults may also opt to commit suicide for their children’s benefit.
Liu (2013)***	Mixed methods	Twenty villages in Hubei, Henan, and Hebei Provinces, China	Key informant interview and questionnaire	Key informants (neighbors, relatives) of 150 suicides in older adults	Suicide among rural older people is associated with intolerable pain caused by physical and mental illness, unfulfilled basic needs, intra-family interpersonal conflicts, neighborhood conflicts, loss of value in life, unfulfilled emotional needs, motivation to lessen the burden on children, and the responsibility to care for relatives. Notably, the distribution of suicide in older adults is influenced by the specific social structure and regional culture of different areas.
Liu et al. (2018)*****	Quantitative nonrandomized	Twenty five towns in Shandong Province, China	Key informant interview and questionnaire	Key informants (close relatives, close friends) of 104 cases of suicide in older adults and 86 nonolder suicide cases, living control persons (*n* = 190)	Compared with the control group, older individuals who died by suicide were more prone to experiencing negative life events and did not live with a spouse. Additionally, they were more likely to have a lower family economic status, engage in farming as an occupation, suffer from physical illness, and have inadequate social support.
Lv et al. (2003) **	Quantitative descriptive	One hundred and forty five villages in Changsha city, Hunan province, China	Key informant interview and questionnaire	Key informants (close relatives, neighbors, remote relatives) of 134 cases of suicide in older adults	The main causes of suicide among the 134 cases include family conflicts, economic hardships, physical illness, and mental or psychological disorders.
Wang et al. (2015)****	Quantitative nonrandomized	Six counties in Shandong Province, China	Face-to-face interview and questionnaire	Suicide attempters (*n* = 365), control persons (*n* = 365)	The brain-derived neurotrophic factor 196G/G genotype was significantly related to attempted suicide in older adults.
Wei et al. (2020)*****	Quantitative nonrandomized	Twelve counties of Shandong, Hunan, and Guangxi Provinces, China	Key informant interview and questionnaire	Key informants (relatives, close friends, colleagues, neighbors) of 242 cases of suicide in older adults (*n* = 484), living control persons (*n* = 242)	When compared with their living counterparts, older adults who died by suicide were more likely to be unemployed, live alone, have an unstable marital status, encounter economic hardship, suffer from physical or mental illnesses, have a family history of suicide, and have experienced significant numbers of stressful life events.
Xia & Xu (2015)***	Qualitative	Six villages in Hubei Province, China	Key informant interview, filed note, official statistics, secondary data from previous literature	Key informants (neighbors, relatives) of 128 cases of suicide in older adults	Suicide in older adults primarily stems from the challenges of accessing necessities and medical care. Instances have been observed where older individuals, unable to endure abuse from their offspring, have opted to end their lives. Furthermore, some older individuals have resorted to suicide to ease the burden they believe they pose on their children.
Yang & Fan (2009)****	Qualitative	Jiufang village in Hubei Province, China	Key informant in-depth interview	Key informants (neighbors) of 24 cases of suicide in older adults	Five factors contribute to the high rates of suicide among older adults in rural China, including the intolerable dullness and solitude of life, the failure to obtain basic food and care, the physical and mental maltreatment from children, exhaustion resulting from poor relationships with children, and the motivation to lessen the burden on children and extend their lifespan.
Yang (2013) ****	Mixed methods	Four rural villages (Feng, Tao, Cha, Dai) in Southern China	Key informant semistructured interview, questionnaire	Key informants (family members, close relatives) of suicide cases (*n* = 114), suicide attempters in older adults (*n* = 19)	Despair is the predominant cause of suicide among older people, with 90% of cases attributed to this emotion, possibly arising from the lack of basic sustenance provided by children, the abuse inflicted by their offspring, or a lack of respect shown by children.
Yang & Ou (2013)****	Mixed methods	Eight rural villages in Central China	Key informant interview, semistructured interview, and official statistics	Key informants (family members, close relatives, neighbors) of cases of suicide in older adults (*n* = 145)	The main factors contributing to the suicide rates among older adults can be identified as follows: conflicts with children, persistent feelings of loneliness, excruciating pain resulting from various illnesses, lack of support from their offspring, and motivation to alleviate the burdens placed upon children.
Zhu et al. (2021)*****	Quantitative nonrandomized	Twelve counties of Shandong, Hunan, and Guangxi Provinces, China	Key informant interviews, questionnaire survey	Key informants (family members, neighbors, remote relatives) of 242 cases of suicide in older adults, living control persons (*n* = 242)	Research indicates a significant association between suicide among older adults, both through violent and nonviolent means, and various factors including mental health disorders, depression, feelings of loneliness, insufficient social support, hopelessness, impulsivity, and a higher number of negative life events. For violent suicide, an additional risk factor was the absence of pesticides in the home. Nonviolent suicide was further associated with being unmarried, having a family history of suicide, and experiencing alcohol-abuse.

*Note*: For study quality, the symbols ^*^, ^**^, ^***^, ^****^, and ^*****^ refer to scores of 20%, 40%, 60%, 80%, and 100%, respectively, obtained on the Mixed Methods Appraisal Tool (MMAT), version 2018; the study design was categorized according to this criterion presented in the MMAT, version 2018.

### Data Synthesis

The included studies encompassed various research methods, including quantitative, qualitative, and mixed-method studies. In this study, a thematic analysis method was employed for data synthesis (Dixon-Woods et al., 2005). This method allows the identification of prominent themes relevant to the review’s aim and offers a structured framework for organizing the literature (Thomas & Harden, 2008). The thematic analysis method consisted of three steps (Thomas & Harden, 2008). In the first step, the included articles were thoroughly reviewed and analyzed, identifying initial codes about the risk factors for suicide among older adults in rural China. In the second step, these codes were grouped into higher-order subthemes and key themes. Finally, with a further conceptualization of the materials, subthemes and key themes were established in the last step. To ensure methodological rigor, the researchers reviewed all the included literature and resolved any coding differences through discussion until a consensus was reached (Hammersley, 1993). Furthermore, during coding, the codes were expanded and modified to ensure their thoroughness and exhaustiveness (Sproule & Walter, 2006). The PRISMA checklist is provided in Supplementary Material 3 (Moher et al., 2009).

## Results

Table 3 presents the characteristics and quality assessments of 16 included studies (Bai et al., 2022; Chen, 2008, 2009; Chiu al., 2012; Li, 2017; Li et al., 2011; Liu, 2013; Liu et al., 2018; Lv et al., 2003; Wang et al., 2015; Wei et al., 2020; Xia & Xu, 2015; Yang, 2013; Yang & Fan, 2009; Yang & Ou, 2013; Zhu et al., 2021). These 16 studies were published between 2003 and 2021. Of the 16 included studies, 4 studies (25%) had a qualitative design, 7 (44%) had a quantitative design, and 5 (31%) had a mixed methods design. For research setting, 10 studies (63%) were conducted in Central China, 6 (38%) in East China, 4 (25%) in South China, 2 (13%) in North China, and 1 (6%) in Northeast China. Four studies spanned regions, and none studies were conducted in Southwest China or Northwest China. The MMAT findings revealed that the quality of the included studies was various, with a mixture of studies having 100% (*n* = 4), 80% (*n* = 6), 60% (*n* = 5), and 40% (*n* = 1) quality. The results revealed that the risk factors for suicide among rural older people were composed of the following six themes.

### Navigating the Challenges of Illness

The most commonly mentioned information regarding the risk factors for suicide among older adults in rural China is the challenges posed by illness. This theme was addressed by 14 articles (Bai et al., 2022; Chen, 2008, 2009; Chiu et al., 2012; Li et al., 2011; Liu, 2013; Liu et al., 2018; Lv et al., 2003; Wang et al., 2015; Wei et al., 2020; Xia & Xu, 2015; Yang & Fan, 2009; Yang & Ou, 2013; Zhu et al., 2021). Two subthemes were identified within this theme: challenges posed by physical illness and those posed by mental illness.

#### Challenges of physical illness

Among older people in rural China, the challenges posed by physical illness are the most common risk factors for suicide. Many illnesses, such as hepatitis, gastric ulcer, and angina, cause great pain to older adults, making some individuals commit suicide as they cannot bear the suffering (Chen, 2008; Chiu et al., 2012). Moreover, asthma, cancer, and other chronic illnesses progressively impair the physical function of rural older individuals, causing substantial inconvenience and distress and severely reducing their quality of life (Liu, 2013; Lv et al., 2003; Yang & Fan, 2009). Consequently, some older persons do not wish to endure a low-quality life, thereby opting to end their suffering through suicide (Liu, 2013; Lv et al., 2003; Yang & Fan, 2009). Studies have supported the importance of physical illness as a risk factor for suicide among older adults in rural China. In a postmortem study of 242 older adults who died by suicide, Bai et al. (2022) revealed that 83.5% of the cases (202 of 242) had a physical illness, which was identified as the most important risk factor by informants. Furthermore, Chiu et al. (2012) and Li et al. (2011) demonstrated a strong association between physical illness and suicides in older individuals in rural China. Additional paired case–control studies have noted a significant association between physical illness and suicide death in rural China. Older adults who died by suicide were more likely to have a physical illness than living controls, and a significant difference in suicide rate between the two groups was found (Liu et al., 2018; Wei et al., 2020).

#### Challenges of mental illness

Mental illnesses, such as depression and anxiety, significantly affect the occurrence of suicide among older persons in rural China (Li et al., 2011; Wei et al., 2020). These illnesses are often accompanied by many symptoms, such as severe stress, deep sadness, helplessness, and mania, inflicting immense pain and suffering on older patients (Li et al., 2011; Wei et al., 2020). Consequently, many older people choose to commit suicide to escape this overwhelming anguish (Lv et al., 2003; Yang & Ou, 2013). Thus, mental illness is crucial in precipitating suicide in rural older adults (Yang & Fan, 2009). Many studies have highlighted that mental illness and suicide are strongly correlated (Li et al., 2011; Wang et al., 2015). In a postmortem survey conducted across three Chinese provinces, more than 50.4% (122 of 242) of older individuals who died by suicide had received a diagnosis of mental disorders from psychiatrists (Bai et al., 2022). Furthermore, Liu et al. (2018) reported that mental disorders were prevalent among suicides within rural older people, and a significant positive correlation existed between the presence of mental disorders and an increased suicide risk.

### Unmet Basic Needs

Nine studies highlighted unmet basic needs as a major risk factor for suicide among rural older people (Chen, 2008, 2009; Li, 2017; Liu, 2013; Lv et al., 2003; Xia & Xu, 2015; Yang, 2013; Yang & Fan, 2009; Yang & Ou, 2013). Two subthemes underpinned this theme: unmet needs for basic dietary intake and those for medical services.

#### Unmet needs for basic dietary intake

Sufficient dietary intake is fundamental for any individual’s survival and well-being. However, older people in rural China are often unable to obtain an adequate amount of food, which can ultimately drive them to commit suicide (Chen, 2008; Liu, 2013; Lv et al., 2003). Unlike urban older persons, rural older adults in China are ineligible for government pensions and typically receive only minimal government subsidies (approximately 15–30 USD/month; Xia & Xu, 2015; Yang, 2013). These subsidies are insufficient for purchasing sufficient food and nutritional products (Xia & Xu, 2015; Yang, 2013). Consequently, unmet basic dietary needs result in a state of chronic hunger, eventually pushing older people toward suicidal thoughts and actions (Li, 2017; Xia & Xu, 2015; Yang, 2013). Older people who are unable to work are especially vulnerable as they no longer earn income through farming, which significantly increases their risk of suicidal tendencies (Chen, 2009; Yang & Fan, 2009; Yang & Ou, 2013). According to the quotes from a qualitative study,

Uncle Wang passed away at the age of seventy. He had been in good health before, able to support himself by growing vegetables. But later he became too weak to work, and found it hard to feed himself. In despair, he drank pesticide and ended his life. (Yang & Ou, 2013).

#### Unmet needs for medical services

As individuals age, their bodies experience deterioration, which consequently leads to an increased risk of various diseases. Therefore, older adults must have access to adequate medical services. However, the lack of such services has created a distressing scenario in the rural areas of China, where a substantial number of older individuals resort to suicide as a means to end their lives (Chen, 2009; Liu, 2013; Xia & Xu, 2015; Yang & Ou, 2013). Liu (2013) found that the lack of medical resources in 20 villages posed significant obstacles in meeting the basic medical needs of older adults. This inadequacy thus plays a noteworthy role as a precipitating factor for suicide in older adults (Liu, 2013). Receiving high-quality care services in rural areas remains challenging, particularly for older adults with physical or cognitive impairments. This impedes their ability to live comfortably and with dignity. Consequently, many older persons ultimately choose voluntary departure from life (Yang & Fan, 2009). Although some older adults can rely on their offspring for care, the majority of them encounter a lack of assistance from children. This can further contribute to their inclination toward suicidal behavior (Xia & Xu, 2015). The qualitative findings of Yang and Ou (2013) corroborated this view,

An old lady in our village suffered from a chronic illness and had to stay in bed. However, her children did not want to take care of her, and she couldn’t receive proper medication and medical care. She often said ‘Life is not interesting’ and soon committed suicide.

### Experiencing Abuse From Children

Abuse experienced by older people in rural China from their adult children significantly and negatively affects their vulnerability to suicide. In other words, when older adults are abused by children, they are more likely to choose to end their own lives. This theme emerged from six studies (Chen, 2009; Li, 2017; Xia & Xu, 2015; Yang, 2013; Yang & Fan, 2009; Yang & Ou, 2013) and encompasses two subthemes: physical abuse by children and emotional abuse by children.

#### Experiencing physical abuse from children

Studies have suggested that physical abuse by children, such as beating, kicking, force-feeding, and binding, has a crucial role in triggering suicide among older adults in rural China (Yang, 2013). Many suicides in rural China occur owing to physical abuse by children, particularly through beating. When older individuals cannot bear the physical abuse inflicted on them, they resort to suicide to seek relief (Li, 2017). Chen (2009) qualitatively demonstrates that several older individuals who committed suicide were physically abused by their offspring. As a quote shows,

An older couple in the village endured the abuse of their ungrateful son. Their younger son frequently beat them cruelly. The two old people, feeling hopeless and desperate, decided to end their lives by suicide.

Meanwhile, most Chinese parents believe that they should make significant sacrifices for their children and hope that their children will reciprocate their love and care. Thus, when they are maltreated by their offspring, they may resort to suicide in anger and psychological imbalance (Yang & Fan, 2009; Yang & Ou, 2013). Furthermore, Chinese rural areas have experienced a decline in the traditional values of filial piety since the 1990s. This cultural shift has alarmingly increased the incidents of disrespect, neglect, and even abuse toward older parents. Consequently, the suicide rate among older people in rural areas has increased significantly (Xia & Xu, 2015; Yang & Ou, 2013).

#### Experiencing emotional abuse from children

Apart from physical abuse, emotional abuse (e.g., insults, curses, scolding, and belittling) also negatively affects suicide rates among older individuals in rural China. Emotional abuse caused by children can lead to significant emotional, cognitive, and behavioral changes, and contribute to the development of depression, anxiety, and eventual suicide (Li, 2017; Yang, 2013). Through their qualitative study, Yang and Fan (2009) revealed that offspring-induced emotional maltreatment is responsible for >20% of cases where older adults choose to commit suicide. With the decrease in the filial piety value in rural areas, verbal maltreatment toward older parents has become prevalent, increasing the suicide risk among parents (Chen, 2009; Xia & Xu, 2015). Some offspring even resort to cursing their parents, which significantly increases the likelihood of older parents committing suicide. As an informant interviewee said in Yang and Ou (2013)’s study,

A middle-aged man in our village placed a bottle of pesticide next to his gravely ill mother and said, ‘When will you die? How can I work out if you don’t die?’ Shortly after, this old woman chooses to leave this world by ingesting the pesticide.

### Feeling of Loneliness

Loneliness is an increasingly prevalent concern among older adults in rural China. It is also significantly associated with suicide among these individuals (Bai et al., 2022). Overall, 56% of the included studies (9 of 16) identified loneliness as a contributing risk factor (Bai et al., 2022; Chen, 2009; Chiu et al., 2012; Li, 2017; Liu et al., 2018; Wei et al., 2020; Yang & Fan, 2009; Yang & Ou, 2013; Zhu et al., 2021). Adding to this issue, the trend of young people migrating from rural to urban areas in search of employment opportunities has led to a rapid increase in the number of “empty nesters”—older parents left behind in rural houses (Li, 2017; Yang & Fan, 2009). Consequently, these older adults often experience feelings of isolation and loneliness. Additionally, bereavement and divorce could also contribute to older adults living alone (Chiu et al., 2012; Liu et al., 2018). This state of living alone exacerbates their feelings of loneliness even more. As a result, many older individuals choose to end their lives because of the overwhelming burdens of loneliness and boredom (Chiu et al., 2012; Li, 2017; Liu et al., 2018; Yang & Fan, 2009). According to a survey conducted across eight villages, the percentage of older adults who resorted to suicide owing to loneliness increased from 6.25% in 1989 to 35.3% in 2009 (Yang & Ou, 2013). In addition, it has been suggested that living without a spouse, whether single, windowed, or divorced, was found to be independently associated with an increased risk of experiencing suicidal thoughts or engaging in suicidal behaviors (Chiu et al., 2012; Liu et al., 2018). Notably, Wei et al. (2020) reported that 26.4% of older individuals who died by suicide lived alone, compared with only 14.5% of the control group, with a statistically significant difference in suicide rates between the two groups. The combination of living alone and experiencing profound loneliness may amplify the distress felt by older adults to the point where life becomes unbearable, leading them to opt for suicide as a viable solution (Chen, 2009; Yang & Fan, 2009).

### Negative Life Events

Negative life events, including a multitude of stressful events and life changes in family life, are also considered significant factors contributing to suicide among older adults in rural China (Bai et al., 2022; Li, 2017; Li et al., 2011; Liu, 2013; Liu et al., 2018; Lv et al., 2003; Wang et al., 2015; Wei et al., 2020; Yang, 2013; Zhu et al., 2021). In many instances, suicide is preceded by exposure to adverse life events (Li et al., 2011; Lv et al., 2003). Wang et al. (2015) and Wei et al. (2020) suggested that negative life events and higher suicide levels among older people in rural China are correlated. Among older individuals, the most prevalent negative life event leading to suicide is the suicide death of a family member. The impact of having a family history of suicide on the suicide rates of rural older adults has been extensively documented (Li et al., 2011; Liu et al., 2018; Zhu et al., 2021). This is expected, particularly within the rural regions of China, where familial bonds are given great importance. A scenario such as a child being diagnosed as having cancer could potentially trigger an older parent to commit suicide (Bai et al., 2022). Furthermore, conflicts within the neighborhood are a prevalent factor triggering suicide among older individuals (Li et al., 2011; Liu, 2013; Lv et al., 2003). In some cases, older adults, experiencing bullying or disputes within their communities, have resorted to suicide as a resolution for their anger (Liu, 2013; Yang, 2013). Lastly, conflicts arising from property rights disputes during the process of housing demolition and relocation have been considered a contributor to the suicides of rural older persons (Li, 2017).

### Altruistic Motivation to Benefit Children

The final theme examined rural older adult’s altruistic motivation to benefit children. This theme was analyzed across seven studies (Bai et al., 2022; Chen, 2009; Li, 2017; Liu, 2013; Xia & Xu, 2015; Yang & Fan, 2009; Yang & Ou, 2013). Two subthemes were identified within this theme: altruistic motivation to reduce children’s financial burden and altruistic motivation to extend children’s lives.

#### Altruistic motivation to reduce children’s financial burden

Some older individuals voluntarily decide to end their own lives, not because of challenges and suffering, but rather because of a desire to not be a burden on their children. For instance, some older adults who are afflicted with illnesses opt to cease living because of the exorbitant medical expenses associated with their illnesses, which can impose a heavy financial burden on their offspring for support (Bai et al., 2022; Li, 2017; Xia & Xu, 2015). Similarly, evidence suggests that some rural older people, recognizing their inability to earn money and their reliance on their children for support, opt to voluntarily depart from life (Chen, 2009; Liu, 2013; Yang & Fan, 2009). Of note, the aforementioned older adults have dutiful and supportive children who are ready to cover their medical and living expenses. Therefore, the decision to commit suicide is not driven by despair, but rather by selfless and altruistic motives (Bai et al., 2022; Chen, 2009; Li, 2017; Liu, 2013; Xia & Xu, 2015; Yang & Fan, 2009). To further exemplify this motivation among older people, a specific case is demonstrated as follows:

Uncle Li suffered from heart disease, and his son was very filial and spent a lot of money on his father’s medical treatment. The old man was very distressed by this. One day he heard that being hit by a car could result in a large sum of compensation. Soon after that, he threw himself in front of a large truck on the highway. His death earned his son forty thousand yuan (Yang & Fan, 2009).

#### Altruistic motivation to extend children’s lives

In rural areas, some older people choose to end their own lives based on a superstitious belief called “*Keshou*” in Chinese. According to this belief, the suicide of older parents increases the lifespan of their adult children (Chen, 2009; Liu, 2013; Yang & Fan, 2009). Specifically, some rural older adults believe that their longevity would deplete the “life force” of their offspring. Consequently, they voluntarily decide to end their lives once they are of a certain age (Chen, 2009). Additionally, some elder people believe that their demise prevents the untimely death of their offspring, leading them to choose to end their lives (Liu, 2013). Moreover, in some instances, rural older adults strongly believe in the notion that their self-inflicted death can transfer their remaining “lifespan” to their descendants, compelling them to prematurely terminate their existence (Yang & Fan, 2009).

## Discussion

**Figure 2.**
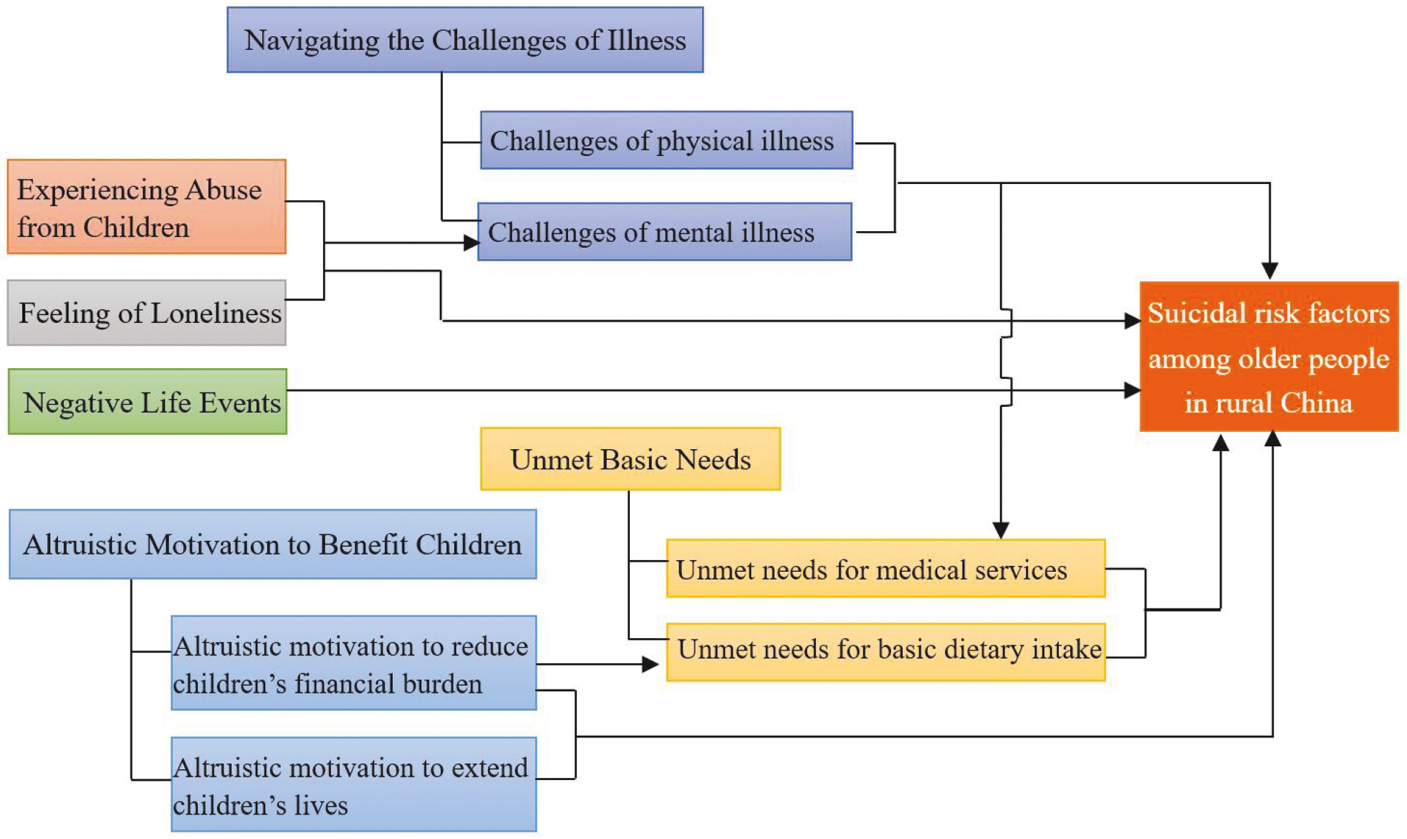
Conceptual framework explaining relationship between themes.

To effectively prevent suicide among older individuals in rural China, a comprehensive understanding of the associated risk factors must be gained. In total, 16 articles were systematically examined to identify relevant risk factors associated with suicide among older people. Suicide among older people has been linked to various risk factors, including illness-related challenges, unmet basic needs, mistreatment by children, loneliness, negative life events, and altruistic motivation to benefit children. The conceptual framework illustrating the relationship between themes can be seen in Figure 2.

First, this study highlighted the impact of illness-related challenges on suicide rates among older individuals. This finding is consistent with the global prevalence of suicide in older people (Harwood et al., 2006; Schmutte & Wilkinson, 2020). Older people are more prone to various physical and mental health conditions than younger individuals (Juurlink et al., 2004). The distress and burden of these conditions often act as risk factors contributing to self-destructive tendencies (Waern et al., 2002). The physical illnesses that contribute to suicide among older people vary across different countries and regions. For instance, in China, rural older adults are influenced by ailments such as cancer, asthma, hepatitis, gastric ulcers, and angina (Chen, 2008; Chiu et al., 2012; Lv et al., 2003; Yang & Fan, 2009). On the other hand, stroke was found to be significantly associated with suicide among older people in Sweden, Denmark, and Japan, whereas kidney problems were identified as a risk for suicide among older individuals in Canada (Fässberg et al., 2016). This disparity could be attributed to the different prevailing types of diseases affecting suicide among older adults in various countries and regions (Beghi et al., 2021). In terms of mental illnesses, depression, and anxiety are the primary mental health disorders contributing to suicide among older people in many countries and regions, including the United States, China, and South Korea (Kułak-Bejda et al., 2021; Li et al., 2011; Wei et al., 2020; Yang & Ou, 2013). Considering the high prevalence of diseases among older individuals in rural areas, they unsurprisingly face the highest risk of suicide (Casant & Helbich, 2022; Chen, Giles, et al., 2022; Zhao et al., 2022).

Second, suicide among older adults in rural China is also linked to unmet basic needs. This may be unexpected considering China’s economic development and agricultural productivity. However, a prevalent phenomenon of “generational exploitation” in rural China can explain this situation. In this situation, parents exhaust their economic resources for the benefit of their children, but the children refuse to support their parents in later years (Deng, 2019). In the absence of financial assistance from their offspring, older people in rural China experience poverty, food insecurity, and inadequate access to healthcare services. Consequently, many of these older people resort to self-inflicted death (Liu, 2013; Yang, 2013). Unmet healthcare needs are associated with higher levels of suicidal behavior among older prisoners (Opitz-Welke et al., 2019), older homeless adults (Kaplan et al., 2019), and older adults with dementia (Alphs et al., 2016). However, scientific evidence concerning the connection between unmet dietary needs and suicide among older people in other parts of the world is lacking. Thus, it is suggested to address the necessity for further research in this domain.

Third, the abuse of elder people by children, encompassing physical and emotional abuse, also contributes to suicide in rural areas. Similar occurrences have been observed in both urban and rural locales in other countries and regions (Osgood & Manetta, 2001; Yunus et al., 2019). All types of older abuse, such as physical, psychological, sexual, and emotional abuse or negligent care, are risk factors for suicide (de Mendonça Lima et al., 2021; Pérez Barrero, 2012). Wand et al. observed that elder abuse can cause harm or distress to older individuals, leading to suicidal behavior, even within trusted relationships (Wand et al., 2018). In rural China, elder abuse significantly originates from family members, particularly sons and daughters (Chen, 2009; Yang & Ou, 2013). Older adults in rural China value family relationships, especially their relationships with children. Hence, when maltreated by their children, the older parents endure intense pain and hopelessness, ultimately seeking solace through suicide (Yang & Fan, 2009; Yang & Ou, 2013). This phenomenon reflects the erosion of filial values and the deterioration of the economic status of older people (Chen, 2009; Yang, 2013).

Fourth, loneliness has been significantly associated with suicide in older adults across most included studies. Globally, several studies have consistently revealed higher levels of loneliness among older individuals who died by suicide (Shoib et al., 2023; Stickley & Koyanagi, 2016). Older adults experiencing social isolation and related loneliness are more likely to have deficient emotion regulation, which in turn increases their suicide risk (De Leo, 2022; Turvey et al., 2002). Consequently, older adults more susceptible to loneliness, such as those who have lost their spouses or live alone, are also at a higher suicide risk (Fernandez-Rodrigues et al., 2022; Heuser & Howe, 2019). In rural China, the migration of children has a significant impact on family relationships. The “empty nesters,” who have lost contact with their children, are particularly vulnerable to the feeling of loneliness (Li, 2017; Yang & Fan, 2009). Currently, neither familial nor societal support has effectively mitigated the loneliness experienced by these older adults (Zheng et al., 2022). As a result, the isolation faced by “empty nesters” in rural China should be emphasized, as it highlights the need for improved social service systems and interventions in caring for these individuals.

Fifth, negative life events (e.g., suicide of family members, neighborhood conflicts) are significant factors contributing to suicide behavior among older adults in rural China. These findings align with those of studies on older adults conducted in other countries globally. In Singapore, adverse life occurrences such as gambling and interpersonal relation disturbance have been found to have a positive correlation with suicide among older adults (Ho et al., 2014). In Finland, familial discord has been identified as a significant adverse life event associated with suicidal behavior in older individuals (Heikkinen & Lönnqvist, 1995). In the United States, negative incidents leading to suicidal behavior among older people encompassed issues with intimate partners, legal troubles, and job problems (Chen & Roberts, 2021). The diverse social contexts encountered by older individuals in different countries and regions result in a variety of adverse life events that can lead to suicide (Conejero et al., 2018).

Lastly, motivation among the older adults of rural China to benefit children could potentially result in suicide behavior, which is not commonly observed among older people in other countries and districts. Some older adults in rural China voluntarily choose to commit suicide to alleviate children’s financial burden and prolong children’s lives. This finding could be interpreted on the basis of Durkheim’s social theory on suicide, which classifies suicide patterns into three categories: egoistic, altruistic, and anomic (Stack, 2004). In the altruistic type, suicide is committed in situations where there is excessive integration into a group and is supported by public opinion (Conejero et al., 2018). This applies to older adults in rural China as well. In the rural areas of China, older people have a strong sense of integration and consider perpetuating the family line as their life’s purpose. Consequently, they prioritize children’s development and well-being over their own lives. This explains their willingness to improve the well-being of children through suicide. This is also accountable for their motivation to improve children’s benefits through self-inflicted death (Li, 2017; Yang & Fan, 2009). Furthermore, the rural Chinese population interprets the self-annihilation of older people for this purpose as a virtuous deed of devotion, which inadvertently encourages this type of suicidal behavior among older people (Chen, 2009; Yang & Ou, 2013). Although it is considered common and understandable in rural China, suicide in older adults therefore appears to be absent in the literature from other nations and regions.

### Knowledge Gaps and Directions for Future Research

This review did not concentrate on personality traits, a factor frequently explored in the study of suicide in older adults (O’Connor & Nock, 2014; Schmutte & Wilkinson, 2020). Early life adversities, including poverty, hunger, and limited access to education, are prevalent among older people in rural China. Additional studies are warranted to explore the effect of these life conditions on suicide risk within this population. Moreover, alcohol and drug use/dependence have been identified as common factors contributing to suicide among older adults in other parts of the globe (Murphy, 2002; Waern, 2003), investigating whether these factors play a similar role in the suicide cases of older people of rural China is crucial. Risk factors may differ between older men and women (Salib & Green, 2003), and thus, further research is required to examine the factors that influence suicide among older men and women in rural China separately.

### Strength and Limitation

To the best of our knowledge, this is the first systematic review examining the risk factors for suicide among older individuals in rural China. The methodology employed had several strengths. Our search method was robust and designed to yield a comprehensive search of the published articles. By covering medical, psychological, and sociological databases and both English and Chinese studies, we could evaluate a diverse range of evidence regarding suicide among older people in rural China. Furthermore, this review followed the PRISMA 2020 guidelines (Moher et al., 2009). The screening, extraction, and coding processes were independently conducted by two researchers, who applied measures to minimize bias (Hammersley, 1993). All studies (Table 3) included in this review were of reasonable-to-good quality on average, with some studies being excellent. This indicates that the effect sizes observed were robust and not influenced by lower-quality studies (Teti et al., 2014).

The review is not without limitations. First, although this was a rigorous systemic literature review, it may not have fully captured all risk factors for suicide among older people in rural China. As with all literature reviews, 10 electronic databases rather than all electronic databases were used for the literature search because of the huge cost associated with the searching of all databases (Bettany-Saltikov, 2016). Therefore, some relevant studies might have been overlooked in this review. Second, this review defines “older adults” as individuals aged ≥60 years, which aligns with the situation in China (Zheng & Wang, 2020). However, this definition may vary across different cultures. At last, the scope of this review was limited to rural China, and therefore, the findings may not be generalized to other regions or countries.

## Conclusion

Suicide among older people in rural China is a significant public health concern. This review found that multiple factors place older adults at-risk for suicide, highlighting the significance of prioritizing prevention and intervention efforts. Recognizing that early detection and prevention are important, healthcare professionals must integrate questions related to suicidal risk factors into routine medical history assessments for rural older adults. Moreover, Chinese governments, nonprofit organizations, and families must collaborate to provide tailored services that increase social and community support for vulnerable rural older people. Finally, our review findings emphasize that improved social and medical welfare programs are required, particularly for older adults in rural areas who face financial hardships.

## Supplementary Material

igae015_suppl_Supplementary_Material

## Data Availability

The original contributions presented in the study are included in the article/Supplementary Material. Further inquiries can be directed to the corresponding author.
