# Supplementary material for: Understanding Risk Factors for Suicide Among Older People in Rural China: A Systematic Review
Source: Innov Aging. 2024 Feb 17;8(3):igae015. doi: 10.1093/geroni/igae015 (PMC11010312; doi:10.1093/geroni/igae015)
Supplement: igae015_suppl_Supplementary_Material [file igae015_suppl_supplementary_material.docx]

***Innovation in Aging* Supplementary Material: Quan Zhang, Shenao Li, & Yijin Wu. Understanding Risk Factors for Suicide Among Older People in Rural China: A Systematic Review.**

**Supplementary Material 1 The literature search terms (MeSH terms) for each database**

**PubMed**

((((((elderly) OR (elder)) OR (aged)) OR (older)) OR (senior)) OR (old)) AND (((((suicide) OR (suicides)) OR (suicidal)) OR (self-murder)) OR (self-slaughter)) AND ((((((((rural) OR (countryside)) OR (village)) OR (peasant)) OR (farmer)) OR (farmers)) OR (villager)) OR (villagers)) AND ((China) OR (Chinese))

**Embase**

(elderly OR elder OR aged OR older OR senior OR old) AND (suicide OR suicides OR suicidal OR self-murder OR self-slaughter) AND (rural OR countryside OR village OR peasant OR farmer OR farmers OR villager OR villagers) AND (China OR Chinese)

**PsycINFO**

("elderly" OR "elder" OR "aged" OR "older" OR "senior" OR "old") AND ("suicide" OR "suicides" OR "suicidal" OR "self-murder" OR "self-slaughter") AND ("rural" OR "countryside" OR "village" OR "peasant" OR "farmer" OR "farmers" OR "villager" OR "villagers") AND ("China" OR "Chinese")

**Cochrane**

(elderly OR elder OR aged OR older OR senior OR old) AND (suicide OR suicides OR suicidal OR self-murder OR self-slaughter) AND (rural OR countryside OR village OR peasant OR farmer OR farmers OR villager OR villagers) AND (China OR Chinese)

**CINAHL**

("elderly" OR "elder" OR "aged" OR "older" OR "senior" OR "old") AND ("suicide" OR "suicides" OR "suicidal" OR "self-murder" OR "self-slaughter") AND ("rural" OR "countryside" OR "village" OR "peasant" OR "farmer" OR "farmers" OR "villager" OR "villagers") AND ("China" OR "Chinese")

**ScienceDirect**

(elderly OR elder OR aged) AND (suicide OR suicides) AND (rural OR countryside) AND (China OR Chinese)

**Web of Science**

TS=((suicide OR suicidal OR suicides OR self-murder OR self-slaughter) AND (rural OR countryside OR village OR peasant OR farmer OR farmers OR villager OR villagers) AND (China OR Chinese) AND (Elderly OR elder OR aged OR older OR senior OR old))

**CNKI**

SU='农村'+'农民'+'村' and SU='自杀'+'轻生'+'死亡' and SU='老年人'+'老人'

**CQVIP**

M=(农村 OR 农民 OR 村) AND M=(自杀 OR 轻生 OR 死亡) AND M=(老年人 OR 老人)

**Wanfang**

题名或关键词: (("农村" or "农民" or "村") and ("自杀" or "轻生"or "死亡") and ("老年人" or "老人"))

**Supplementary Material 2 Mixed Methods Appraisal Tool (MMAT), version 2018**

**1. Qualitative**

Q1. Is the qualitative approach appropriate to answer the research question?

Q2. Are the qualitative data collection methods adequate to address the research question?

Q3. Are the findings adequately derived from the data?

Q4. Is the interpretation of results sufficiently substantiated by data?

Q5. Is there coherence between qualitative data sources, collection, analysis and interpretation?

**2. Quantitative randomized controlled trials**

Q1. Is randomization appropriately performed?

Q2. Are the groups comparable at baseline?

Q3. Are there complete outcome data?

Q4. Are outcome assessors blinded to the intervention provided?

Q5. Did the participants adhere to the assigned intervention?

**3. Quantitative non-randomized studies**

Q1. Are the participants representative of the target population?

Q2. Are measurements appropriate regarding both the outcome and intervention (or exposure)?

Q3. Are there complete outcome data?

Q4. Are the confounders accounted for in the design and analysis?

Q5. During the study period, is the intervention administered (or exposure occurred) as intended?

**4. Quantitative descriptive studies**

Q1. Is the sampling strategy relevant to address the research question?

Q2. Is the sample representative of the target population?

Q3. Are the measurements appropriate?

Q4. Is the risk of nonresponse bias low?

Q5. Is the statistical analysis appropriate to answer the research question?

**5. Mixed methods**

Q1. Is there an adequate rationale for using a mixed method design to address the research question?

Q2. Are the different components of the study effectively integrated to answer the research question?

Q3. Are the outputs of the integration of qualitative and quantitative components adequately interpreted?

Q4. Are divergences and inconsistencies between quantitative and qualitative results adequately addressed?

Q5. Do the different components of the study adhere to the quality criteria of each tradition of the methods involved?

**Quality appraisal of included studies (Mixed Methods Appraisal Tool scores)**

| Author, Year | Study design | Q1 | Q2 | Q3 | Q4 | Q5 | Comments | MMAT score |
| --- | --- | --- | --- | --- | --- | --- | --- | --- |
| Bai et al. (2022) | Mixed methods | √ | √ | √ | -- | √ | Mixed methods is an appropriate method to address the study question, and it was properly applied in the researching process. However, the integration of the qualitative and quantitative components was not adequately interpreted. | 80% |
| Chen (2008) | Qualitative | √ | √ | √ | × | × | The data collected are rich and sufficient. However, the data collection and data analysis process were implicit in the paper and is not explicitly stated. The links between data sources, collection, analysis, and interpretation are unclear. | 60% |
| Chen (2009) | Mix method | √ | √ | √ | √ | × | Mixed methods is an appropriate method to address the study question, and the research findings of this paper are rich and profound. However, the methodology section of this paper is overly simplistic and lacks a clear explanation of data collection and analysis process. | 80% |
| Chiu et al. (2012) | Quantitative non-randomized | √ | √ | √ | × | -- | The study followed a classical analytical method of a quantitative study. The sampling method is rational, variable measurement and data analysis followed a scientific process. However, the confounders seem to be somewhat limited. | 60% |
| Li et al. (2011) | Quantitative non-randomized | √ | √ | √ | × | -- | The study followed a classical analytical method of a quantitative study. However, the confounders were not accounted for in the design and analysis. | 60% |
| Li (2017) | Qualitative | √ | √ | √ | √ | √ | Qualitative method is appropriate to address the study question, and it was was executed with scientific rigor. The process of data collection, analysis, and interpretation adhered to a scientific methodology. Furthermore, clear coherence was found between qualitative data sources, collection, analysis and interpretation | 100% |
| Liu (2013) | Mixed methods | √ | √ | √ | -- | × | Mixed methods was generally properly applied throughout the research process, and the qualitative and quantitative components of the study were effectively integrated to answer the research question. However, the methods for data collection and analysis could have been more clearly articulated. | 60% |
| Liu et al. (2018) | Quantitative non-randomized | √ | √ | √ | √ | √ | The study adhered to the rigorous analytical process typical of a quantitative study. The procedures for sampling, data collection, variable measurement, and data analysis were scientifically conducted. Furthermore, the research findings of this paper are both rich and profound, contributing significantly to the field. | 100% |
| Lv et al. (2003) | Quantitative descriptive | √ | √ | -- | × | × | The sampling method of this study was properly utilized; thus, the sample appears to be representative of the target population. However, the reliability and validity of the variable measurement tools were not clearly reported, which is a crucial aspect of any quantitative study. Furthermore, the findings could have been more detailed and profound to provide a deeper understanding of the research topic. | 40% |
| Wang et al. (2015) | Quantitative non-randomized | √ | √ | × | √ | √ | A stringent analytical process characteristic of a quantitative study was followed in the study. The methods for sampling, data collection, variable measurement, and data analysis were conducted in a scientific manner. However, the findings seem simplistic and not profound enough. | 80% |
| Wei et al. (2020) | Quantitative non-randomized | √ | √ | √ | √ | √ | The study followed a stringent analytical process characteristic of a quantitative study. The findings of this paper are both rich and profound, making a significant contribution to the field. | 100% |
| Xia & Xu (2015) | Qualitative | √ | -- | √ | √ | -- | It seems the study failed to have the analytic sharpness needed in qualitative content analysis. The methods of data collection and data analysis process were not clearly stated. The links between data sources, collection, analysis, and interpretation are unclear. | 60% |
| Yang & Fan (2009) | Qualitative | √ | -- | √ | √ | √ | The study followed a classical analytical method of quantitative study. The findings of this paper are rich and profound. However, the data collection and data analysis process were implicit in the paper and is not explicitly stated. | 80% |
| Yang (2013) | Mix method | √ | √ | √ | -- | √ | Mixed methods is an appropriate method to address the study question, and it was properly applied in the researching process. The qualitative and quantitative components of the study were effectively integrated to answer the research question. However, the sampling method of this study could have been more clearly articulated. | 80% |
| Yang & Ou (2013) | Mix method | √ | √ | √ | √ | -- | The utilization of mixed methods was aptly suited to address the research question and was effectively implemented throughout the study. The findings of this paper are rich, novel, and insightful, thereby making a substantial contribution to the discipline. However, the methodologies employed for sampling and analysis were not clearly delineated. | 80% |
| Zhu et al. (2021) | Quantitative non-randomized | √ | √ | √ | √ | √ | The study adhered to a rigorous analytical procedure typical of a quantitative study. The findings of this paper are both abundant and insightful, making a notable contribution to the field. | 100% |

Note: Q1, Q2, Q3, Q4, Q5 refers to the question number of the scales. Studies of different design types was appraised with different scales shown above. √refers to “Yes”, × refers to “No”, -- refers to “Can’t tell”.

**Supplementary Material 3 PRISMA 2020 Checklist of this systematic review**

| **Section and Topic** | **Item #** | **Checklist item** | **Location where item is reported** |
| --- | --- | --- | --- |
| **TITLE** | | |  |
| Title | 1 | Identify the report as a systematic review. | Page 1 |
| **ABSTRACT** | | |  |
| Abstract | 2 | See the PRISMA 2020 for Abstracts checklist. | Page 2 |
| **INTRODUCTION** | | |  |
| Rationale | 3 | Describe the rationale for the review in the context of existing knowledge. | Page 4-5 |
| Objectives | 4 | Provide an explicit statement of the objective(s) or question(s) the review addresses. | Page 6 |
| **METHODS** | | |  |
| Eligibility criteria | 5 | Specify the inclusion and exclusion criteria for the review and how studies were grouped for the syntheses. | Page 7 |
| Information sources | 6 | Specify all databases, registers, websites, organisations, reference lists and other sources searched or consulted to identify studies. Specify the date when each source was last searched or consulted. | Page 7 & Table 2 |
| Search strategy | 7 | Present the full search strategies for all databases, registers and websites, including any filters and limits used. | Page 7, Table 1 & Supplementary Material 1 |
| Selection process | 8 | Specify the methods used to decide whether a study met the inclusion criteria of the review, including how many reviewers screened each record and each report retrieved, whether they worked independently, and if applicable, details of automation tools used in the process. | Page 7-8 |
| Data collection process | 9 | Specify the methods used to collect data from reports, including how many reviewers collected data from each report, whether they worked independently, any processes for obtaining or confirming data from study investigators, and if applicable, details of automation tools used in the process. | Page 8 |
| Data items | 10a | List and define all outcomes for which data were sought. Specify whether all results that were compatible with each outcome domain in each study were sought (e.g. for all measures, time points, analyses), and if not, the methods used to decide which results to collect. | NA |
|  | 10b | List and define all other variables for which data were sought (e.g. participant and intervention characteristics, funding sources). Describe any assumptions made about any missing or unclear information. | NA |
| Study risk of bias assessment | 11 | Specify the methods used to assess risk of bias in the included studies, including details of the tool(s) used, how many reviewers assessed each study and whether they worked independently, and if applicable, details of automation tools used in the process. | Page 8 |
| Effect measures | 12 | Specify for each outcome the effect measure(s) (e.g. risk ratio, mean difference) used in the synthesis or presentation of results. | NA |
| Synthesis methods | 13a | Describe the processes used to decide which studies were eligible for each synthesis (e.g. tabulating the study intervention characteristics and comparing against the planned groups for each synthesis (item #5)). | NA |
|  | 13b | Describe any methods required to prepare the data for presentation or synthesis, such as handling of missing summary statistics, or data conversions. | NA |
|  | 13c | Describe any methods used to tabulate or visually display results of individual studies and syntheses. | NA |
|  | 13d | Describe any methods used to synthesize results and provide a rationale for the choice(s). If meta-analysis was performed, describe the model(s), method(s) to identify the presence and extent of statistical heterogeneity, and software package(s) used. | Page 9 |
|  | 13e | Describe any methods used to explore possible causes of heterogeneity among study results (e.g. subgroup analysis, meta-regression). | NA |
|  | 13f | Describe any sensitivity analyses conducted to assess robustness of the synthesized results. | NA |
| Reporting bias assessment | 14 | Describe any methods used to assess risk of bias due to missing results in a synthesis (arising from reporting biases). | NA |
| Certainty assessment | 15 | Describe any methods used to assess certainty (or confidence) in the body of evidence for an outcome. | NA |
| **RESULTS** | | |  |
| Study selection | 16a | Describe the results of the search and selection process, from the number of records identified in the search to the number of studies included in the review, ideally using a flow diagram. | Figure 1 |
|  | 16b | Cite studies that might appear to meet the inclusion criteria, but which were excluded, and explain why they were excluded. | NA |
| Study characteristics | 17 | Cite each included study and present its characteristics. | Page 9-10 & Table 3 |
| Risk of bias in studies | 18 | Present assessments of risk of bias for each included study. | Supplementary Material 2 |
| Results of individual studies | 19 | For all outcomes, present, for each study: (a) summary statistics for each group (where appropriate) and (b) an effect estimate and its precision (e.g. confidence/credible interval), ideally using structured tables or plots. | Page 10-17 |
| Results of syntheses | 20a | For each synthesis, briefly summarise the characteristics and risk of bias among contributing studies. | NA |
|  | 20b | Present results of all statistical syntheses conducted. If meta-analysis was done, present for each the summary estimate and its precision (e.g. confidence/credible interval) and measures of statistical heterogeneity. If comparing groups, describe the direction of the effect. | NA |
|  | 20c | Present results of all investigations of possible causes of heterogeneity among study results. | NA |
|  | 20d | Present results of all sensitivity analyses conducted to assess the robustness of the synthesized results. | NA |
| Reporting biases | 21 | Present assessments of risk of bias due to missing results (arising from reporting biases) for each synthesis assessed. | NA |
| Certainty of evidence | 22 | Present assessments of certainty (or confidence) in the body of evidence for each outcome assessed. | NA |
| **DISCUSSION** | | |  |
| Discussion | 23a | Provide a general interpretation of the results in the context of other evidence. | Page 17-21 |
|  | 23b | Discuss any limitations of the evidence included in the review. | Page 21-22 |
|  | 23c | Discuss any limitations of the review processes used. | Page 22 |
|  | 23d | Discuss implications of the results for practice, policy, and future research. | Page 3 & Page 23 |
| **OTHER INFORMATION** | | |  |
| Registration and protocol | 24a | Provide registration information for the review, including register name and registration number, or state that the review was not registered. | NA |
|  | 24b | Indicate where the review protocol can be accessed, or state that a protocol was not prepared. | NA |
|  | 24c | Describe and explain any amendments to information provided at registration or in the protocol. | NA |
| Support | 25 | Describe sources of financial or non-financial support for the review, and the role of the funders or sponsors in the review. | Page 23 |
| Competing interests | 26 | Declare any competing interests of review authors. | Page 24 |
| Availability of data, code and other materials | 27 | Report which of the following are publicly available and where they can be found: template data collection forms; data extracted from included studies; data used for all analyses; analytic code; any other materials used in the review. | Page 24 |
